# Patients with cyclops syndrome exhibit elevated synovial fluid interleukin‐6 (IL‐6) and tumor necrosis factor alpha (TNF‐α) levels and disorganized collagen remodelling within the lesion: A histological and molecular study

**DOI:** 10.1002/jeo2.70953

**Published:** 2026-10-17

**Authors:** Luca Morici, Philippe M. Tscholl, Eline. L. Skau, Klaus Eyer, Lukas Clerc, Morgan Gauthier, Ines Nikolic

**Affiliations:** ^1^ Institute of Pharmaceutical Sciences of Western Switzerland University of Geneva Geneva Switzerland; ^2^ Department of Orthopaedics and Trauma Surgery University Hospital of Geneva Geneva Switzerland; ^3^ Department of Biomedicine, Faculty of Health Sciences Aarhus University Aarhus Denmark

**Keywords:** ACL reconstruction, arthrofibrosis, cyclops lesions, cyclops syndrome, IL‐6, single‐cell analysis

## Abstract

**Purpose:**

Cyclops syndrome is a clinically relevant complication of anterior cruciate ligament (ACL) reconstruction characterized by the formation of a fibrotic nodule (cyclops lesion) that causes extension loss and often requires a revision surgery. Biological mechanisms underlying lesion formation remain poorly understood. The purpose of this study is to characterize the tissue architecture, cellular composition, inflammatory profile and single‐cell cytokine secretion patterns of patient‐derived biopsies to gain mechanistic insight into disease pathogenesis.

**Methods:**

This prospective observational comparative study included three patient groups: patients undergoing revision surgery for cyclops syndrome (*n* = 10), patients undergoing primary ACL reconstruction with subsequent successful recovery (*n* = 10) and patients undergoing revision surgery for ACL graft re‐rupture (*n* = 2). Tissue biopsies and synovial fluid samples were collected intraoperatively and used for histological, cellular and cytokine analyses.

**Results:**

Histological analyses demonstrated that cyclops lesions exhibited disorganized collagen architecture, neovascularization and infiltration of immune cells. Flow cytometry identified activated FAP^+^CD54^+^ fibroblasts as the predominant cell population (~80%), accompanied by CD206^+^ pro‐fibrotic macrophages. Synovial fluid from cyclops patients contained significantly elevated IL‐6 and TNF‐α concentrations, whereas IL‐1β levels did not differ among the groups. Looking at the cells, single‐cell cytokine secretion profiles revealed detectable IL‐6 secretion in up to 11% of cells, while IL‐1β, TNF‐α and TGF‐β secretion were not detected, indicating that at least the source of TNF‐α has to be found elsewhere.

**Conclusions:**

Our preliminary data from a small patient cohort show that cyclops syndrome may be observed as an active fibro‐inflammatory process rather than a purely mechanical post‐operative complication. Patient‐derived samples revealed persistent inflammatory activity characterized by immune cell infiltration, elevated levels of pro‐inflammatory mediators, and sustained activation of fibroblasts. The absence of detectable TGF‐β secretion despite established fibrosis suggests that lesion persistence may be maintained through alternative fibrotic pathways (non‐TGF‐β/non‐canonical pathways), which requires further investigation.

**Level of Evidence:**

N/A.

## Graphical abstract

A stepwise approach: from tissue biopsies to histological and molecular analysis.

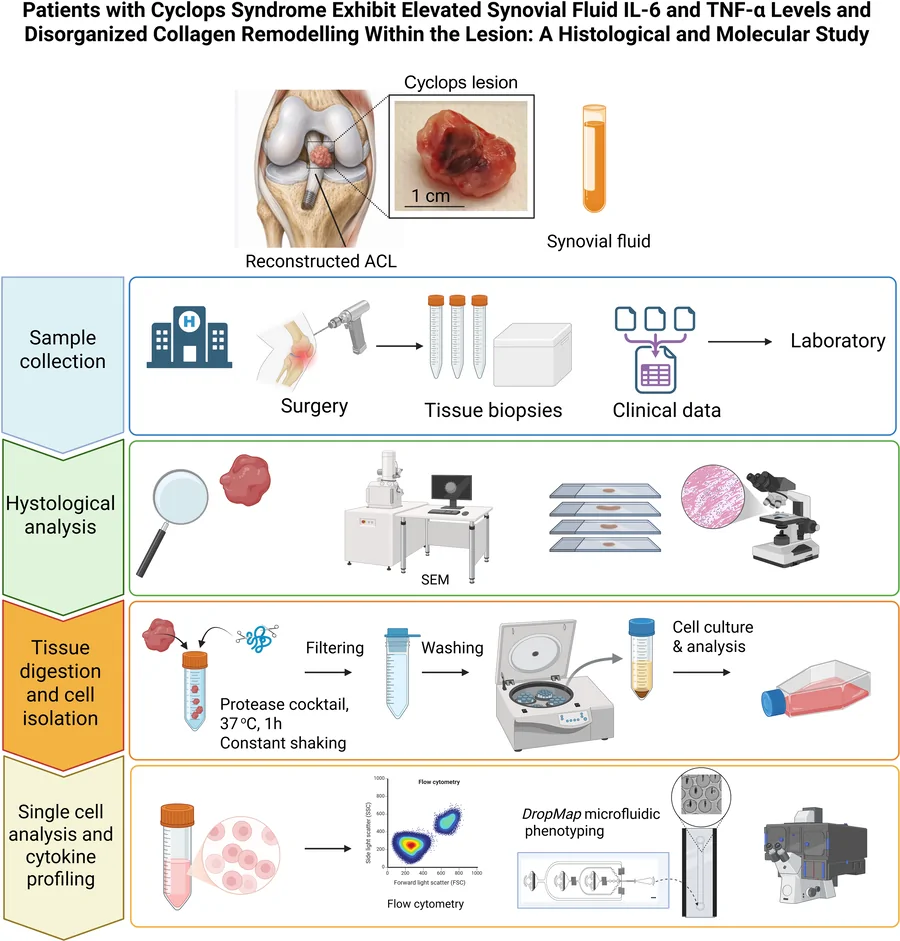

**Abbreviations**
ACL: anterior cruciate ligament
FAP: fibroblast activation protein
H&E: haematoxylin and eosin
IL: interleukin
MT: Masson's trichrome
PBS: phosphate‐buffered saline
PFA: paraformaldehyde
SR: Sirius red
TGF‐β: transforming growth factor‐β
TNF‐α: tumour necrosis factor‐α

## INTRODUCTION

Cyclops syndrome is a form of localized knee anterior arthrofibrosis specifically related to the anterior cruciate ligament (ACL) reconstruction surgery [16, 25]. It is characterized by the formation of a fibrous nodule anterior to the reconstructed ACL (cyclops lesion). Although asymptomatic cyclops lesions are frequently observed following ACL reconstruction, the prevalence of clinically relevant complications (referred to as cyclops syndrome), recognized by significant and persistent leg extension deficits, stiffness and a specific ‘clunk’ sound near full leg extension, usually requiring a subsequent surgical intervention to remove the knee blockage, is around 10% of all ACL reconstruction cases [1, 5, 16, 27, 28]. Several hypotheses have been proposed to explain the formation of cyclops lesions, including microtrauma due to the repetitive mechanical contact between the neoligament and the intercondylar notch [23], reaction to debris from drilling the bone tunnel, native ACL remnants [16], graft size [26] and anterior position of the tibial tunnel [1]. Yet none have been conclusively established.

Despite its impact on patient outcomes and burden for the healthcare system, the pathophysiology of cyclops syndrome remains poorly understood. Current literature is largely focused on retrospective analyses reporting incidence and clinical observations, while mechanistic insights into its development remain limited [3, 8, 41]. In particular, evidence regarding the inflammatory and immune‐mediated pathways underlying this pathological fibrosis remains limited [24, 43].

In the present study, we investigated cyclops syndrome from a translational perspective. We hypothesized that cyclops syndrome results from unresolved post‐operative inflammation, reflecting an active fibro‐inflammatory process rather than a purely mechanical complication of ACL reconstruction. Therefore, the purpose of this study is to characterize the tissue architecture, cellular composition, inflammatory profile and single‐cell cytokine secretion patterns of patient‐derived biopsies to gain mechanistic insight into disease pathogenesis.

## MATERIALS AND METHODS

### Patient population, surgical procedures and sample collection

Tissue samples were collected from patients undergoing revision surgery for cyclops syndrome (*n* = 10), primary ACL reconstruction surgery (Control group 1, *n* = 10), or revision ACL reconstruction due to graft re‐rupture (Control group 2, *n* = 2) at the University Hospital of Geneva. Only patients with isolated ACL injuries were included. Patients with concomitant meniscal or cartilage lesions, as well as other knee injuries or pre‐existing knee pathologies, were excluded. All patients included in both the cyclops and control groups received an autograft harvested from the quadriceps tendon (QT autograft). In all cases, any remnants of the native ACL were completely debrided and removed from the joint cavity to ensure clear visualization and anatomical placement. No native ligament tissue was preserved or utilized in the reconstruction. Specific attention was given to not leaving any torn ligament behind that might have already been in the position of a potential cyclops. The tibial tunnel was placed in an outside‐in manner, with full socket. The aimer was positioned using the ACL stump (before complete debridement) and the anterior horn of the lateral meniscus. The position was validated in near full extension of the knee, not to risk roof impingement. For the first two post‐operative weeks, patients were allowed weight‐bearing at half of their body weight, and then increased as tolerated. Patients were systematically wearing a brace in full extension at night only during the first 2 weeks, to emphasize the importance of full knee extension. Intra‐articular surgical drains were not used in the cohort. None of the patients required subsequent therapeutic joint aspiration for severe post‐operative effusion or re‐operation. Mild post‐operative joint effusion, when present, was managed conservatively with cryotherapy and non‐steroidal anti‐inflammatory drugs.

During primary ACL reconstruction procedures and revision reconstructions due to graft re‐rupture, ACL remnants, graft tissue and synovial fluid (if present) were collected. During revision surgery due to cyclops syndrome, excised fibrotic tissue (cyclops lesions) and synovial fluid (if present) were obtained. All specimens were obtained from tissue routinely discarded during surgery, and no additional procedures were performed for research purposes. Patient characteristics are presented in Table 1. All participants were informed about the objectives of the study and provided written informed consent prior to inclusion. The study was approved by the Cantonal Ethics Committee of Geneva (approval number: 2025‐00617). The study flowchart is presented in Figure 1.

**Table 1:** Baseline demographic characteristics of the study population.

	Patients with cyclops syndrome	Control group 1 (primary ACL reconstruction)	Control group 2 (revision ACL reconstruction due to graft re‐rupture)
Number of patients	10	10	2
Age,^a^ median in years (min–max)	35 (20–47)	24 (20–42)	22 (20–24)
Sex^b^	40% female, 60% male	20% female, 80% male	50% female, 50% male
Time elapsed after ACL reconstruction, median in months (min–max)	6 (4–12)	n.a.	n.a.
Time elapsed between injury and ACL surgery,^c^ median in months (min–max)	2 (1–3)	n.a.	2.5 (1–4)
Prior treatments	None of the included patients had received intra‐articular injections in the period after the ACL reconstruction or prior to the cyclops lesion removal.		
Concomitant knee pathologies/injuries	None	None	None

Abbreviation: ACL, anterior cruciate ligament.

^a^ No statistically significant differences were observed among the groups (Kruskal–Wallis test, *p* = 0.18).

^b^ No statistically significant differences were observed among the groups (Fisher's exact test, *p* = 0.54).

^c^ No statistically significant differences were observed between the groups (Mann–Whitney test, *p* = 0.75).

**Figure 1:**
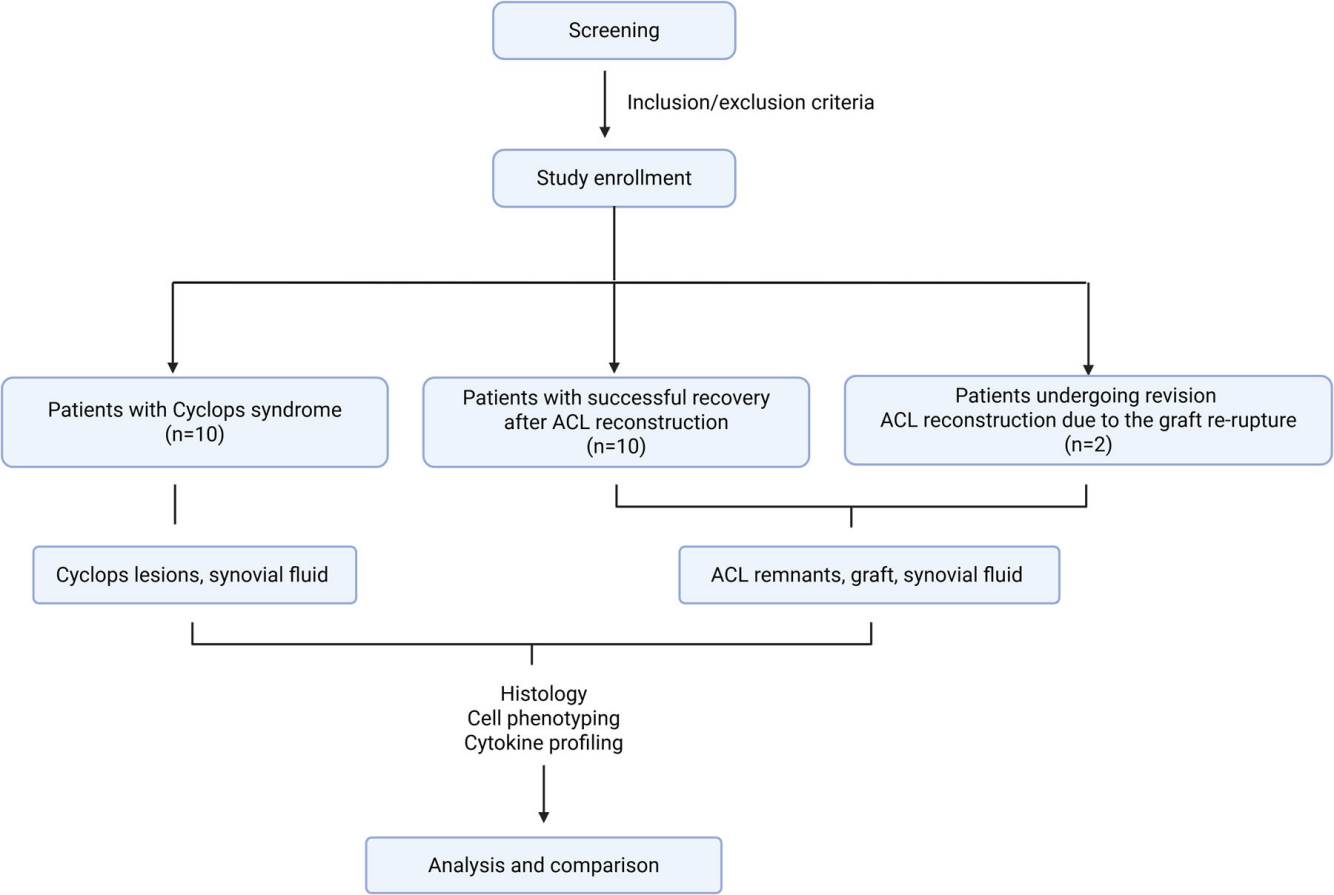
Study flowchart. ACL, anterior cruciate ligament.

In the cyclops group, biopsies from three randomly selected patients were used for histological and microscopic analyses, while the remaining samples underwent cell isolation for further investigations. Synovial fluid was available from four patients in this group. Similarly, in Control group 1 (patients with successful recovery), three tissue biopsies were used for histological and microscopic analyses, while synovial fluid was collected from six patients. In Control group 2 (patients undergoing revision surgery due to the ligament re‐rupture), synovial fluid was available for all patients.

All samples were collected in sterile plastic tubes and transported on ice to the laboratory for immediate processing and analysis. The maximum time from the collection of the tissue biopsies to their arrival at the laboratory for further analysis was 30 min.

As inflammatory markers were assessed, the control groups included only patients undergoing primary ACL reconstruction or ACL reconstruction following ligament re‐rupture performed at least four weeks after injury to allow the acute inflammatory response to subside and avoid introducing bias into the comparative analysis. This criterion was not applied to patients undergoing revision surgery for cyclops syndrome. In addition, all patients in the control groups were enroled in a standard long‐term post‐operative follow‐up programme, and none of the patients developed cyclops syndrome.

### Tissue morphology analysis by scanning electron microscopy (SEM)

To investigate the ultrastructural organization of the tissue samples beyond the resolution achievable by light microscopy, SEM was performed. Following fixation in 4% paraformaldehyde (PFA, Electron Microscopy Sciences) for 8 h in the fridge and washing with phosphate‐buffered saline (PBS, Thermo Fisher Scientific), tissue biopsies were obtained from larger extracted tissue specimens using a dermal punch. Samples were subsequently dehydrated through a graded ethanol series (30, 50, 70, 96 and >99.45% v/v, Fisher Scientific). Tissues were immersed in each ethanol solution for 15 min, except for absolute ethanol (30 min) to ensure complete dehydration, followed by 45 min of drying using an Autosamdri® critical point dryer (Tousimis). Afterwards, samples were mounted onto metallic specimen holders covered with a double‐sided conductive carbon adhesive tape (Science Services). Prior to imaging, samples were sputter‐coated with a 20 nm gold layer using a high‐vacuum sputter coater (Leica Microsystems, Germany) to improve conductivity and minimize charging artefacts during analysis. Samples were then visualized using a field emission scanning electron microscope (JSM‐7001F, JEOL). Images were acquired using the secondary electron detector under high‐vacuum conditions at 5 kV accelerating voltage and a working distance of 10 mm.

### Histological analysis

For histological analysis, tissue samples were fixed in 4% PFA (Electron Microscopy Sciences) at 2–8°C overnight and subsequently washed with sterile PBS. Samples were dehydrated through a graded ethanol series as described above, cleared in xylene (Sigma‐Aldrich), embedded in paraffin (Rectolab), and sectioned into 4–6 µm slices using a microtome Leica RM2265 (Leica Biosystems). Tissue sections were mounted onto glass slides and stained for histological evaluation.

Haematoxylin and eosin (H&E) staining was performed to assess general tissue morphology, cellularity, and structural organization. Sirius red (SR) staining was used to evaluate collagen deposition and connective tissue organization, including collagen fibre distribution under polarized light microscopy. In addition, Masson's Trichrome (MT) staining was performed to further confirm the presence and distribution of collagen‐rich fibrotic connective tissue. Histological images were acquired using a Digital Slide Scanner Axioscan 7 (Carl Zeiss).

### Cell isolation from the tissue biopsies

Tissue digestion was carried out using an enzymatic cocktail consisting of collagenase type I from *Clostridium histolyticum* (2 mg/mL; Sigma‐Aldrich) and dispase II (1 mg/mL; Sigma‐Aldrich) prepared in sterile PBS containing Ca^2+^ and Mg^2+^ (Thermo Fisher Scientific).

Larger tissue samples were cut into smaller fragments and transferred into sterile tubes containing the digestion solution. Enzymatic digestion was performed in a shaker incubator at 37°C for 45 min under constant agitation at 150 rpm. Following digestion, samples were filtered through 70 µm cell strainers (Easy strainers, Greiner Bio‐One) into sterile tubes to remove residual, undigested tissue fragments. Cell suspensions were subsequently diluted with complete Dulbecco's modified Eagle medium (DMEM, Thermo Fisher Scientific) at twice the digestion volume to deactivate enzymatic activity and centrifuged at 400 × *g* for 5 min (Eppendorf Centrifuge 5702, Eppendorf). The supernatant was discarded, and the cell pellet was resuspended in 1 mL of complete DMEM.

Cell number and viability were determined using a Countess™ II FL Automated Cell Counter (Life Technologies, Thermo Fisher Scientific) following Trypan Blue staining.

### Flow cytometry

Flow cytometry was performed to characterize the cellular populations present within the tissue samples using selected surface markers.

The cells were first gated by excluding debris, doublets, and dead cells, using propidium iodide as a viability dye (Ready Flow™, Thermo Fisher Scientific). The following fluorophore‐conjugated antibodies were used for cell phenotyping: CD45 (immune cell marker; BD Biosciences, cat. number: 563880), CD11b (myeloid cell marker; BD Biosciences, cat. number: 555388), CD80 (M1 macrophage‐associated markers BD Biosciences, cat. number: 567442), CD86 (M1 macrophage‐associated marker; BD Biosciences, cat. number: 563411), CD206 (M2 macrophage‐associated marker; Affymetrix eBioscience, cat. number: 53‐2069‐41), CD3 (pan‐T‐lymphocyte marker; BD Biosciences, cat. number: 740832), CD4 (T‐helper lymphocyte marker; BD Biosciences, cat. number: 563876), CD54/ICAM‐1 (inflammation‐associated fibroblast marker; BD Biosciences, cat. number: 770232) and FAP (fibroblast activation protein; activated fibroblast marker, R&D Systems FAB3715R‐100UG). All antibodies were added in the concentrations following the manufacturers' instructions.

The cells isolated from tissue were collected, centrifuged, and the cell pellet was resuspended in 100 µL of FACS buffer (PBS containing 2% FBS). Subsequently, the appropriate volume of antibody was added. The cell suspension was gently vortexed and incubated with antibodies for 20 min at 4°C in the dark. Afterwards, 1 mL of FACS buffer was added, and samples were centrifuged at 400 × *g* for 5 min (Eppendorf Centrifuge 5702, Eppendorf). The supernatant was discarded, and the cells were resuspended in 500 µL of FACS buffer for analysis.

Samples were analyzed using a BD LSRFortessa™ flow cytometer (BD Biosciences) equipped with BD FACSDiva™ software.

### Microfluidic single‐cell phenotyping using *DropMap* technology

For measurement of their cytokine secretion, cyclops lesion‐derived cells were compartmentalized into monodisperse ~50 pL aqueous droplets, isolating the cells from each other. The obtained droplets were then filled immobilized in a two‐dimensional array, which allowed for the longitudinal monitoring of secretion events at the single‐cell level [30, 31]. In this study, the technique was used to characterize the cytokine secretion profiles of cells isolated from cyclops lesions obtained during revision surgery.

All microfluidic consumables were prepared as described elsewhere [21]. Measured cytokines included interleukin (IL)‐1β, tumour necrosis factor‐α (TNF‐α), IL‐6, and transforming growth factor‐β (TGF‐β). Droplets were produced, arrays generated, and images were taken as described in Linder et al. [21] at 37°C and ambient oxygen. Images were acquired every 30 min over a 4‐h period, resulting in 9 time points. Data analysis was performed using a custom MATLAB script as described elsewhere [30]. A schematic overview of the workflow is provided in Figure 2. For more details on the experimental protocol and antibodies, please refer to Table S1.

**Figure 2:**
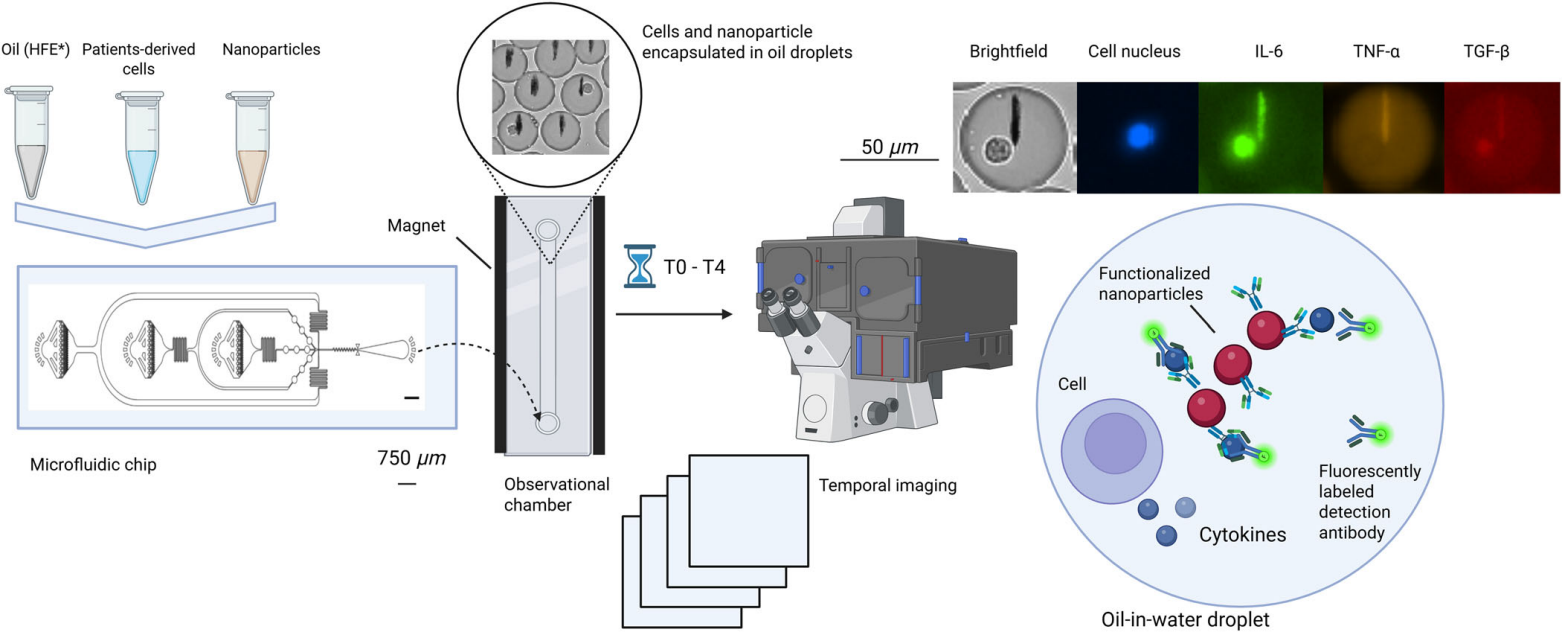
Schematic overview of the microfluidic single‐cell phenotyping using the *DropMap* workflow. Image generated via BioRender.

### Synovial fluid analysis: pH measurements and cytokine profiling

As a simple physicochemical parameter, the pH of synovial fluid was measured by direct immersion of a pH probe (pH metre 827, Metrohm).

Enzyme‐linked immunosorbent assay (ELISA) was used to analyze synovial fluid for IL‐1β and TNF‐α concentrations (RAB0273 and RAB0476 kits, Sigma‐Aldrich), while the IL‐6 assay was performed using a Lumit® IL‐6 (human) Immunoassay (Promega). All assays were conducted according to the manufacturers' instructions.

### Statistical analysis

Data processing and statistical analyses were performed using GraphPad Prism v10.5.1 (GraphPad Software). Data distributions were assessed for normality using the Shapiro–Wilk test prior to statistical testing. Given the small sample sizes and the non‐normal distribution observed for several variables, non‐parametric tests were used where appropriate. Comparisons among the three independent groups were performed using the Kruskal–Wallis test, followed by Dunn's multiple‐comparison test when appropriate. Comparison between two independent groups was performed using the Mann–Whitney test. For categorical variables, such as sex distribution, Fisher's exact test was used. Continuous variables are presented as mean ± standard deviation (SD) for normally distributed data or as median and interquartile range (IQR) otherwise, as indicated in the text and figures. All tests were two‐sided, and *p* < 0.05 was considered statistically significant.

## RESULTS

### Cyclops lesions: Fibro‐inflammatory structures characterized by region‐dependent orientation of collagen fibres and immune cell infiltration

Microscopic analysis of histological sections demonstrated high collagen abundance, with regional variations in structural organization. Figure 3 depicts representative sections of cyclops lesions of all samples subjected to the histology analysis in the cyclops group. SR micrographs observed under bright field (Figure 3a–c) are characterized by dense collagen deposition but without specific fibre orientation. When observed under polarized light (Figure 3d–f), prominent green birefringence could be seen, corresponding to less compact, disorganized, more dispersed fibres characteristic of active healing and fibrosis [18, 22]. MT staining confirmed that collagen lacked a specific orientation pattern and that the tissue was interspersed with prominent neovascularization (Figures 3g–i and 4c). These observations were consistent across all samples. In contrast, peripheral fibres, compared to the central region, appeared better oriented and more mature (Figure 4a), reflecting the chronic evolution and fibroproliferative nature of the cyclops lesion. Furthermore, in some parts of the peripheral areas of cyclops lesion sections, a reduced MT staining intensity was observed, consistent with regions of lower collagen density and a more proteoglycan‐rich extracellular matrix (Figure 4b).

**Figure 3:**
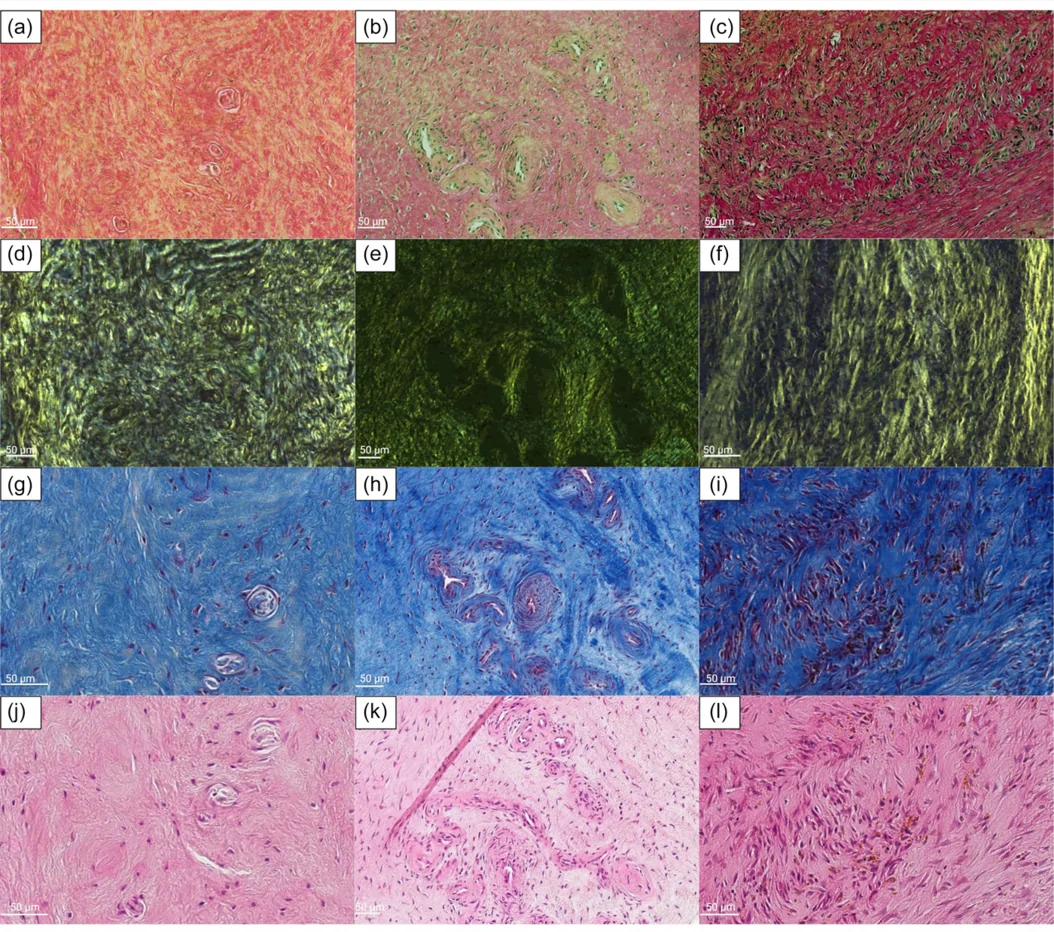
Histological characterization of cyclops lesions from three patients. Each column corresponds to one patient. (a–c) Sirius Red staining under bright‐field illumination showing abundant collagen deposition. (d–f) Corresponding Sirius Red‐stained sections viewed under polarized light, showing predominantly green birefringence consistent with relatively thin and/or less organized collagen fibres. (g–i) Masson's trichrome staining showing disorganized tissue architecture and high cellularity. (j–l) Haematoxylin and eosin staining showing disorganized collagen fibre orientation and numerous fibroblast‐like cells. Magnification: 20×; Scale bars: 50 µm; *n* = 3.

**Figure 4:**
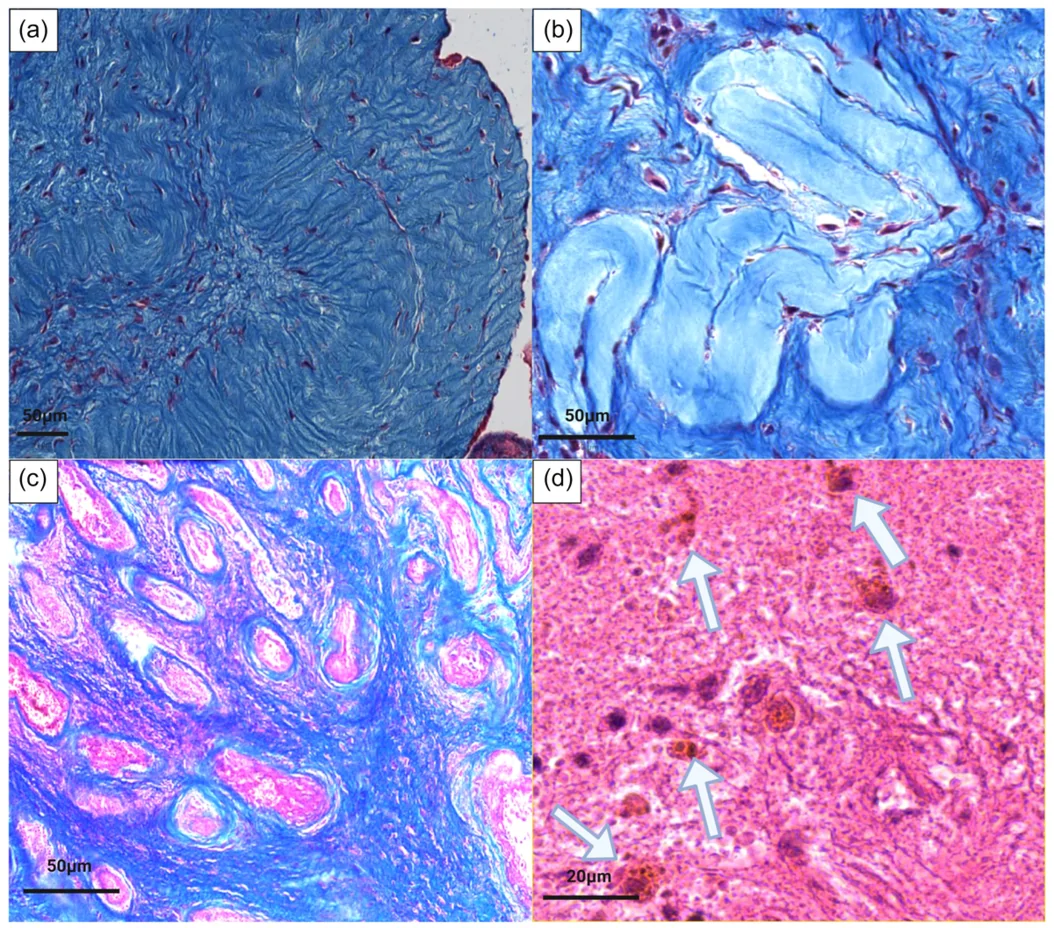
Histological characterization of the peripheral and central regions of cyclops lesions. (a) Masson's trichrome staining of the peripheral region showing more organized and mature collagen fibre architecture. (b) Masson's trichrome staining of the peripheral region showing deposition of a proteoglycan‐rich matrix (light blue). (c) Masson's trichrome staining of the central region showing prominent neovascularization (blue: collagen; pink: blood vessels). (d) Haematoxylin and eosin staining showing hemosiderin‐containing macrophages (white arrows). Magnification: 20×. Scale bars: (a–c) 50 µm; (d) 20 µm.

Observed structural state contrasted sharply with the graft histology, which exhibited the highly organized, tightly packed and wavy structural architecture, with numerous, elongated fibroblasts aligned parallel to the dense collagen fibres (Figure 5a–d). Such an organization was present both at the periphery and in the central region, indicating structural homogeneity which was not observed in cyclops lesions. Consistently, the ACL remnants exhibited a high degree of organization with characteristic wavy collagen bundles (Figure 5e–h). When SR sections were observed under polarized light (Figure 5b,f), yellow birefringence was predominant, corresponding to dense and mature, structured collagen fibres.

**Figure 5:**
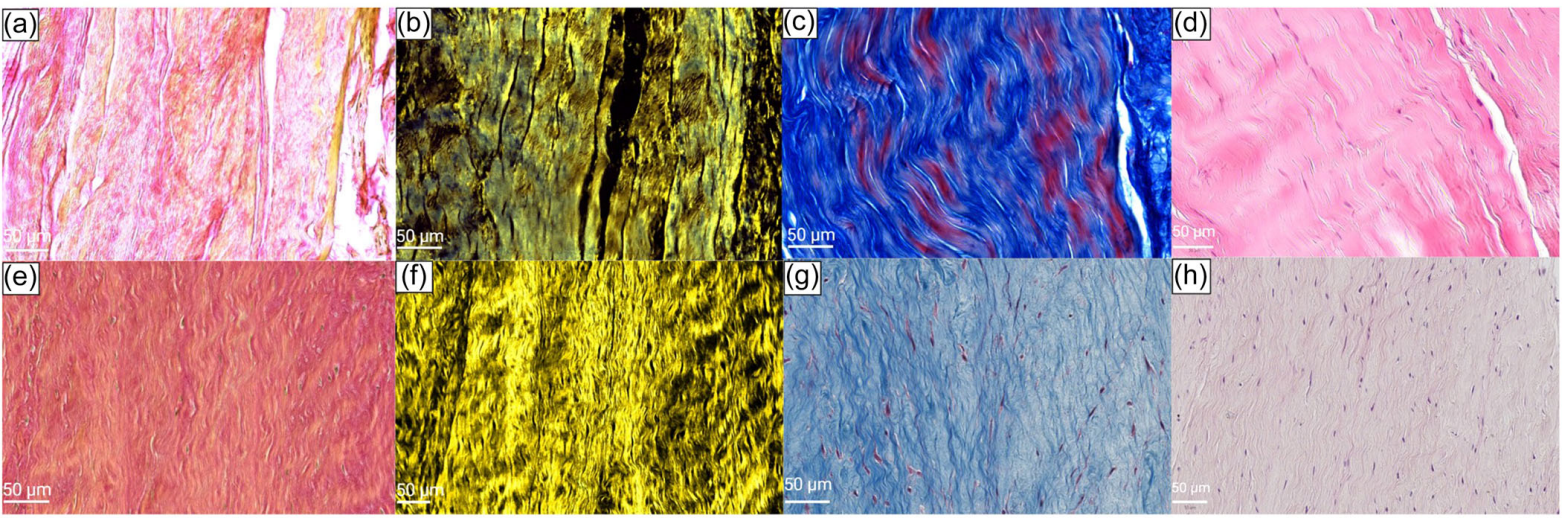
Histological characterization of the tendon graft and native ACL tissue. Representative sections of graft tissue (a–d) and native ACL tissue (e–h) showing well‐organized, parallel collagen fibres and elongated fibroblast‐like cells. (a) Sirius Red staining of the tendon graft tissue under bright‐field illumination, showing collagen‐rich areas (pink/red regions). (b) Sirius Red‐stained section of the tendon graft viewed under polarized light, showing predominantly yellow birefringence consistent with relatively thick and densely organized collagen fibres. (c) Masson's trichrome staining of the tendon graft showing a wavy and well‐organized collagen fibre architecture. (d) Haematoxylin and eosin staining of the tendon graft showing elongated fibroblast‐like cells aligned along the organized collagen fibres. (e) Sirius Red staining of native ACL tissue under bright‐field illumination, showing abundant collagen deposition (red). (f) Sirius Red‐stained section of the native ACL viewed under polarized light, showing predominantly yellow birefringence corresponding to thicker collagen fibres. (g) Masson's trichrome staining of the native ACL showing the characteristic wavy organization of collagen fibres. (h) Haematoxylin and eosin staining of the native ACL showing numerous fibroblast‐like cells along the fibres. Magnification: 20×; Scale bars: 50 µm. ACL, anterior cruciate ligament.

The ‘chaotic’ architecture of the cyclops lesion was further corroborated by SEM, which clearly revealed high tissue porosity (Figure 6a) and a lack of the highly ordered fibrillar hierarchy (Figure 6b) seen in the native ACL (Figure 6c) or tendon graft controls (Figure 6d). More importantly, in cyclops lesion specimens, individual fibres could not be clearly visualized or their structural organization assessed, in contrast to the graft and ACL biopsies, where distinct fibre bundles were readily identifiable.

**Figure 6:**
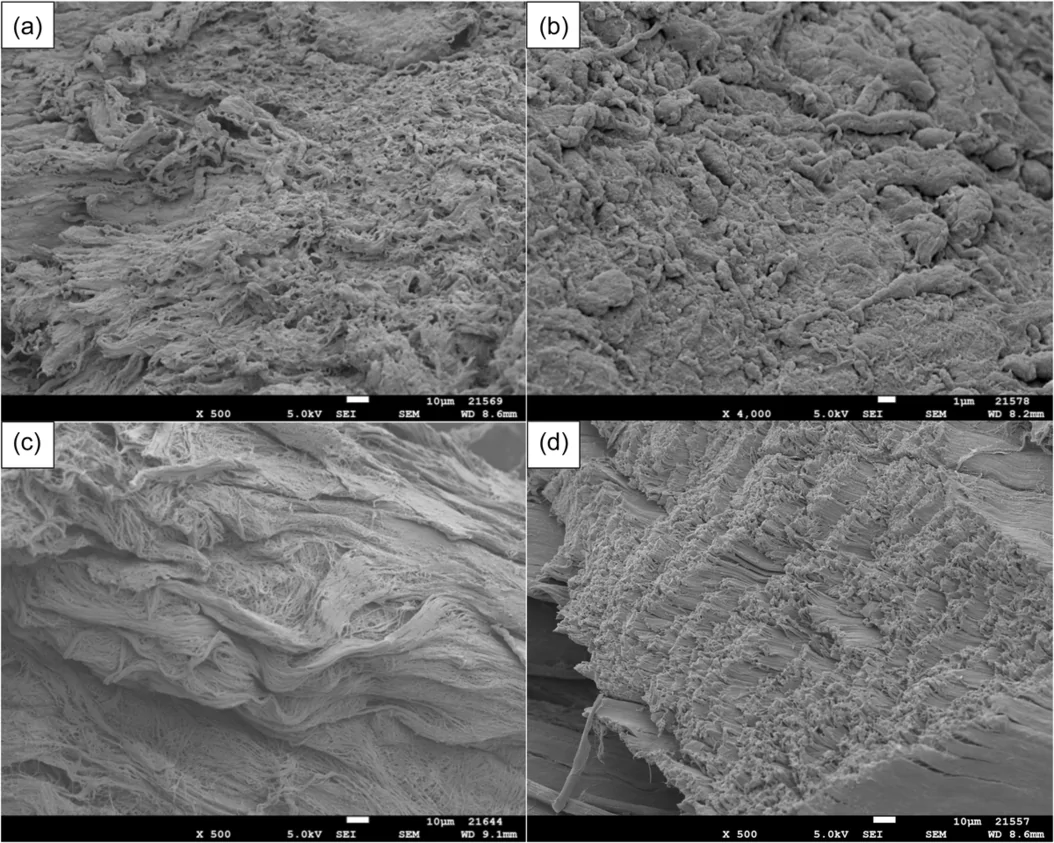
Representative scanning electron microscopy images of cyclops lesions (a, b), native ACL (c) and graft biopsy (d). Scale bars: (a, b, d) 10 µm; (c) 1 µm; *n* = 3 patients per group.

Observing the cell population in the section of the cyclops lesions, in addition to fibroblasts, which were the dominant cellular population (Figure 3j–l), clusters of infiltrating immune cells (macrophages) were easily identified on H&E sections (Figure 4d). These cells were readily distinguished by intracellular accumulations of hemosiderin (golden‐brown colour).

### Cellular characterization reveals predominance of activated fibroblasts in cyclops lesions

In order to identify the cell populations present in the tissue, cells derived from cyclops lesion biopsies were analyzed using flow cytometry (Figure 7). The predominant cell population was composed of fibroblasts (around 80%). In all cases, fibroblasts were characterized by expression of FAP (Figure 7a), indicating a highly activated cellular state consistent with ongoing tissue remodelling and proliferation. Furthermore, in our study, all FAP^+^ cells were also highly CD54^+^ (Figure 7d). Activated fibroblasts are known for high expression of CD54 (Intercellular Adhesion Molecule 1, ICAM‐1), which facilitates their interaction with immune cells [34]. CD54 is broadly expressed on endothelial cells and stromal fibroblasts, particularly under inflammatory conditions, and therefore represents a general marker of cellular activation and inflammatory response (sometimes with bidirectional roles), rather than a lineage‐specific identifier [12, 32].

**Figure 7:**
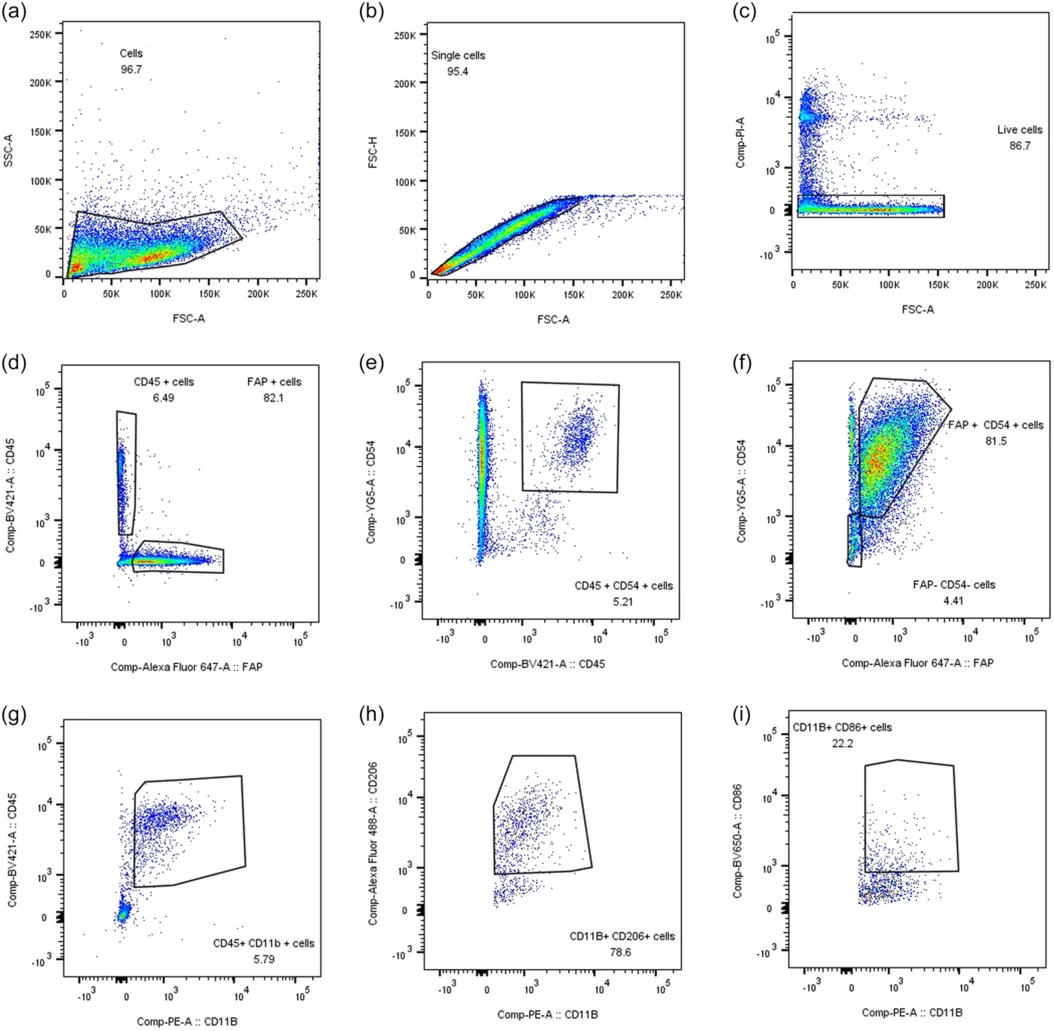
Gating strategy used for the flow cytometric analysis of cyclops lesion‐derived cells. Total cells were identified based on FSC‐A/SSC‐A parameters (a), followed by the selection of single cells (b) and exclusion of PI^+^ (dead) cells (c). CD45^+^ immune cells and FAP^+^ fibroblasts were identified (d). Subpopulation analyses included CD45^+^CD54^+^ cells (e), FAP^+^CD54^+^ cells (f), myeloid cells (CD45^+^CD11b^+^) (g), and myeloid cell subpopulations expressing CD206 (h) or CD86 (i).

In tested samples, there was a small cell population that was CD45^−^FAP^−^CD54^+^. Given the known expression of ICAM‐1 on endothelial cells [19] and the pronounced vascularization of cyclops tissue, this population may in part represent endothelial cells associated with neovessel formation.

In addition, around 10% of all cells (10.62% ± 8.88%) in the tissue were CD45^+^CD11b^+^ (Figure 8), indicating infiltration of myeloid immune cell populations. No significant population of other leucocyte subsets was detected. Interestingly, within the immune cell compartment, the majority of cells were CD206^+^ (Figures 7h and 8). Although this represents a highly simplified classification, these cells may be considered M2‐like, consistent with a pro‐fibrotic macrophage phenotype [29]. This was followed by a small (1%–2%), but still present population of CD86^+^ cells (Figure 7i). Given the absence of the CD80 marker, it may be assumed that the population of CD86^+^ macrophages belongs to the M2b subpopulation [25].

**Figure 8:**
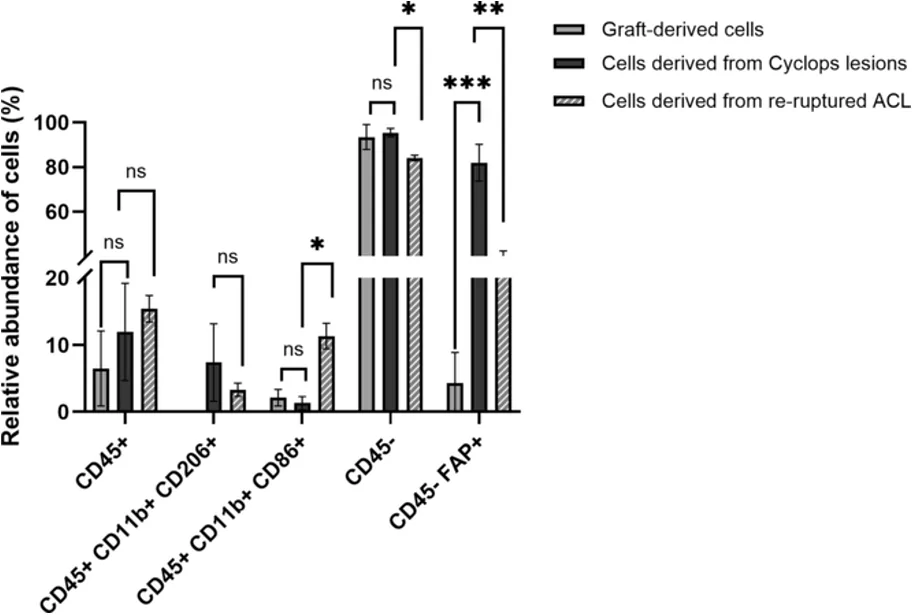
Flow cytometric characterization of cells isolated from tendon graft (*n* = 7), cyclops lesions (*n* = 7) and re‐ruptured ACL (*n* = 2), showing the relative abundance of the main cell populations (mean ± standard deviation) identified based on surface marker expression; Kruskal–Wallis test: statistical significance is indicated as *p* < 0.05 (*), *p* < 0.01 (**), *p* < 0.001 (***), and non‐significant (ns). ACL, anterior cruciate ligament.

In contrast, cells isolated from graft biopsy tissue were predominantly composed of quiescent fibroblasts, with minimal immune cell infiltration, consistent with the cellular composition of healthy tendon tissue (Figures 8 and S1). Similar results were obtained for the ACL remnants (Figure S2).

Importantly, tissue from the already reconstructed ligament showed significantly lower FAP expression compared with cyclops lesion tissue (Figure 8). This finding further supports a more pronounced activated fibroblast phenotype in cells derived from cyclops lesions.

### Synovial fluid of cyclops patients shows high levels of proinflammatory cytokines

As a primary indicator of local characteristics of the altered environment in the knee, the pH value of synovial fluid could be used [11]. However, when values of cyclops and control groups were compared, no significant difference was observed (Figure 9a; *p* = 0.7395). In all cases, the values were slightly alkaline, closer to 8, and for certain patients even higher, which is quite far from the values expected for inflammation‐mediated pathologies [38]. Therefore, in this study, pH did not indicate an altered joint microenvironment.

**Figure 9:**
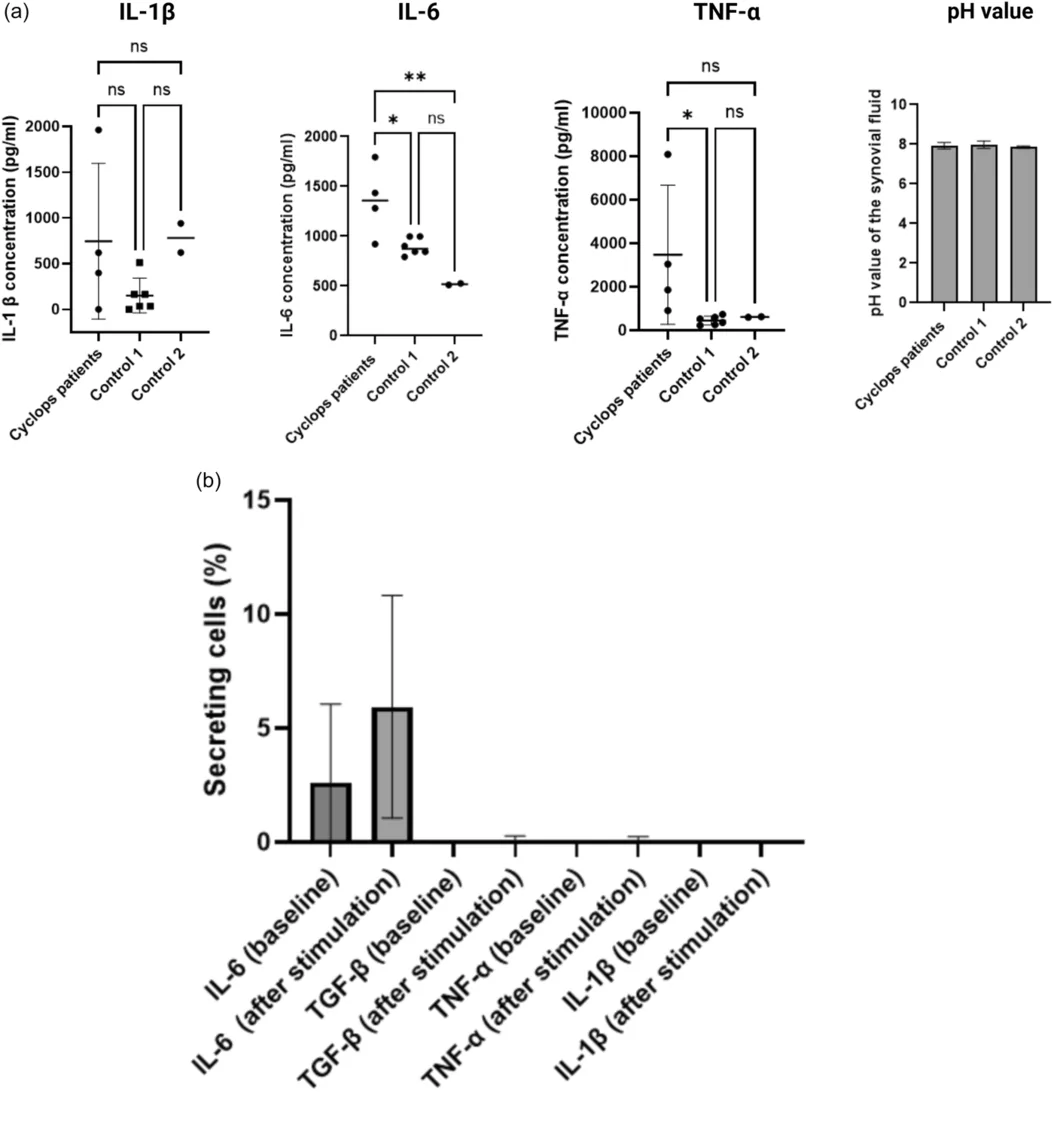
(a) Cytokine profiling and pH measurements in synovial fluid samples; *n* = 4 patients for the cyclops group, *n* = 6 patients for the control group 1 and *n* = 2 for the patients in the control group 2 (patients with ACL re‐rupture). Kruskal–Wallis test: Statistical significance is indicated as *p* < 0.05 (*), *p* < 0.01 (**) and non‐significant (ns). (b) Cytokine secretion by cyclops lesion‐derived cells, expressed as the percentage of cytokine‐secreting cells under basal conditions (baseline) and after stimulation with IL‐1β (50 ng/mL for 4 h); *n* = 3 patients. ACL, anterior cruciate ligament.

All three analyzed cytokines (IL‐1β, IL‐6 and TNF‐α) were detectable at high concentrations in the synovial fluid of patients with cyclops syndrome and in both control groups, although differences in cytokine levels were observed between groups (Figure 9a, Table S2). IL‐6 levels were significantly higher in patients with cyclops syndrome than in both control groups (*p* = 0.0238 vs. control group 1; *p* = 0.0042 vs. control group 2). TNF‐α levels were significantly higher in patients with cyclops syndrome compared with control group 1 (*p* = 0.0195), whereas no significant difference was observed compared with control group 2. However, this result should not be interpreted as evidence of the absence of a biological difference because, due to the very small sample size in control group 2, statistical power is very limited. Finally, no significant differences were observed in IL‐1β levels among the three groups (Figure 9a).

### DropMap microfluidic single‐cell profiling shows IL‐6 plays a critical role in fibroblast‐macrophage crosstalk

Among all investigated cytokines, IL‐6 was the only cytokine secreted by a substantial proportion of cells, reaching up to 7% of the population under basal conditions (Figure 9b). In contrast, TGF‐β, IL‐1β and TNF‐α secretions could not be detected, neither at the baseline conditions nor after stimulation. This result was in a way surprising given the high concentrations of pro‐inflammatory cytokines detected in synovial fluid (Figure 9a), suggesting that inflammation is present but may originate from other surrounding tissues and cells. Following stimulation, the fraction of IL‐6 secreting cells increased to 11%, although not significantly (*p* = 0.09), and these IL‐6‐secreting cells may represent a distinct macrophage subpopulation responding to IL‐1β stimulation (Figure 9b). Notably, TGF‐β remained undetectable, which is of particular interest given its reported role in modulating scarring processes and fibrosis.

## DISCUSSION

The primary results of the study suggest that patients with cyclops syndrome are characterized by a persistent and chronic inflammatory and tissue‐remodelling microenvironment in the knee even months after the initial ACL reconstruction. This was reflected by elevated concentrations of TNF‐α and IL‐6 in synovial fluid.

In addition, IL‐6 was consistently detected at the single‐cell level of cyclops‐derived cells, identifying it as the predominant active inflammatory mediator. Persistent IL‐6 production may indicate activation of the IL‐6/JAK/STAT3 signalling cascade, which promotes the transcription of genes involved in cell survival, proliferation, angiogenesis, and tissue remodelling, including hypoxia‐inducible factor‐1α (HIF‐1α) [20]. HIF‐1α has itself been implicated in the amplification of profibrotic responses and extracellular matrix deposition [25]. This predominance of IL‐6 observed both at the single‐cell level and in synovial fluid, together with TNFα in synovial fluid, is consistent with previous reports demonstrating that IL‐6 and TNF‐α remain elevated for prolonged periods following ACL injury and reconstruction, even after the acute inflammatory phase has subsided [36]. This suggests that persistent low‐grade inflammation may represent an important component of the pathological environment associated with cyclops syndrome.

Flow cytometry revealed a predominance of FAP^+^ fibroblasts, suggesting the presence of an activated fibroblast population, displaying enhanced proliferative and migratory behaviour [4], contributing to the growth of the cyclops lesion. This activated phenotype can be induced by both tissue damage‐associated signals and mechanical stimuli. Following injury and surgery, fibroblasts transition from a quiescent state to a migratory and proliferative phenotype. Alterations in tissue structure and matrix stiffness, together with a physically constrained microenvironment in the knee, further promote fibroblast activation and persistence [6, 7, 10]. Based on a significant body of evidence, FAP has been proposed as a robust indicator associated with pathological tissue remodelling [15]. It is a marker of activated stromal fibroblasts and has also been reported in other contexts of pathological activation, including cancer‐associated fibroblasts [39]. It is a serine protease implicated in extracellular matrix remodelling, with little to no expression in normal fibroblasts [17]. Moreover, FAP^+^ cells were also highly CD54^+^, facilitating the crosstalk with leucocytes. This marker is upregulated in response to inflammatory mediators, including IL‐1β and TNF‐α [33], which were also present in high concentrations in synovial fluid of cyclops patients. However, as FAP is also physiologically essential for the healing process [6], it could be expected that after the ACL reconstruction surgery, FAP is expressed to a high degree in the graft fibroblasts several months after the reconstruction. Nevertheless, our results show that patients undergoing revision surgery due to the re‐rupture of already reconstructed ligament show significantly lower predominance of FAP^+^ fibroblasts, further confirming that cyclops patients actually have prolonged fibroblast activation persisting beyond the healing process. Overall, the extended elevation of analyzed cytokines beyond the expected resolution of post‐operative inflammation may contribute to ongoing fibroblast activation and extracellular matrix remodelling, thereby supporting maintenance of the fibrotic lesion, and contributing to the inflammatory loop without resolution [35].

Additionally, our histology data clearly demonstrate that cyclops lesions are characterized by disorganized collagen architecture where cell proliferation is overdue. Such an organization together with vascular ingrowth present in analyzed specimens, has already been reported [2, 42]. In this context, the proteoglycan‐rich extracellular matrix observed at the periphery of cyclops lesions may reflect a local fibrocartilaginous adaptation to repetitive mechanical compression exerted on the lesion during attempts to achieve full knee extension [9, 37].

FACS analysis pointed out immune cell infiltration, mostly CD206^+^ “pro‐fibrotic”/“anti‐inflammatory” macrophages. These cells are known to promote extracellular matrix deposition, tissue remodelling, and fibrotic responses through their interactions with fibroblasts and secretion of profibrotic mediators. However, persistent activation of ‘anti‐inflammatory’ macrophages, as well as disturbed communication among macrophages, epithelial cells, stem cells and fibroblasts, has also been implicated in pathological fibrosis [44], suggesting that these cells could contribute to the excessive extracellular matrix deposition characteristic of cyclops lesions. Still, based on the overall analysis, it can be assumed that persistent fibroblast activation may not depend solely on inflammatory signalling, but also be a consequence of mechanotransduction pathways triggered by altered extracellular matrix organization and increased tissue stiffness [40, 45]. Finally, it may also be argued that ACL reconstruction can cause a certain biochemical imbalance locally, which, in certain patients, fails to be resolved properly, leading to a fibrotic loop.

Looking further into the secretion profile of single cells, the fact that TGF‐β was undetectable is of particular interest given its reported role in modulating scarring processes and fibrosis. One possible explanation is that a substantial fraction of TGF‐β is secreted and subsequently retained within the extracellular matrix in its latent form [14], making it less accessible to antibody‐based detection approaches that primarily target soluble cytokine fractions, directly secreted from the cells. Furthermore, fibrosis is increasingly recognized as a self‐sustaining process that may persist after the initial TGF‐β stimulus has subsided. Once activated, fibroblasts can maintain a profibrotic phenotype through epigenetic reprogramming, metabolic adaptation and persistent mechanical cues from the surrounding microenvironment, thereby entering a chronic, injury‐independent state [13].

Overall, these findings support the hypothesis that cyclops lesions represent a failure of proper resolution of the post‐surgical inflammatory and remodelling response, characterized by persistent fibroblast activation and sustained immune cell infiltration. The ICAM‐1 expression suggests an inflammatory surrounding environment, but based on the type of detected macrophages, tissue remodelling and fibrosis are predominant, rather than active inflammation.

### Study limitations

This study has several limitations which should be acknowledged. First, the small sample size limits the generation of definitive conclusions, providing only preliminary confirmation of the presented hypothesis. Moreover, patients with arthrofibrosis rarely produce sufficient synovial fluid for sampling, which limited the number of specimens available for synovial fluid analysis. Furthermore, TGF‐β was not quantified in the synovial fluid samples, preventing direct comparison between local cytokine concentrations and single‐cell secretion profiles. Furthermore, a substantial proportion of TGF‐β may be present in the extracellular matrix in its latent form, where it would not be detected by conventional cytokine assays. Future studies should therefore assess both soluble and matrix‐associated latent TGF‐β to better define its contribution to cyclops lesion development. In the context of cell phenotyping, additional surface markers could be introduced in the analysis to obtain a better understanding of different cell subpopulations. Another limitation is that cellular secretion was evaluated under in vitro conditions following cell isolation. The disruption of the native tissue architecture may alter cellular behaviour, particularly for fibroblasts and macrophages whose phenotypes are strongly influenced by cell–cell interactions, extracellular matrix composition, mechanical forces, and oxygen gradients. Three‐dimensional culture systems that better recapitulate the in vivo microenvironment may therefore provide a more physiologically relevant model for investigating the mechanisms underlying cyclops syndrome [25].

## CONCLUSIONS

Our preliminary data from a small patient cohort show that cyclops syndrome may be observed as an active fibro‐inflammatory process rather than a purely mechanical post‐operative complication. Patient‐derived samples revealed persistent inflammatory activity characterized by immune cell infiltration, elevated levels of pro‐inflammatory mediators and sustained activation of fibroblasts. The absence of detectable TGF‐β secretion despite established fibrosis suggests that lesion persistence may be maintained through alternative fibrotic pathways (non‐TGF‐β/non‐canonical pathways), which requires further investigation. Given the very small sample size, these findings should be considered exploratory and interpreted with appropriate caution. Future research should focus on validating these findings in larger patient cohorts and further elucidating the molecular pathways involved in lesion development and persistence.

## AUTHOR CONTRIBUTIONS

**Luca Morici**: Data curation; investigation; formal analysis; visualization; writing—original draft; writing—review and editing. **Philippe M. Tscholl**: Investigation; resources; project administration; supervision; writing—review and editing. **Eline. L. Skau**: Data curation; investigation; formal analysis; writing—review and editing. **Klaus Eyer**: Investigation; formal analysis; visualization; resources; supervision; writing—review and editing. **Lukas Clerc**: Investigation; writing—review and editing. **Morgan Gauthier**: Investigation; writing—review and editing. **Ines Nikolic**: Data curation; investigation; formal analysis; visualization; resources; supervision; project administration; writing—original draft; writing—review and editing.

## CONFLICT OF INTEREST STATEMENT

The authors declare no conflicts of interest.

## ETHICS STATEMENT

This study was approved by the Cantonal Ethics Committee of Geneva (approval number: 2025‐00617). All participants were informed about the objectives of the study and provided written informed consent prior to inclusion.

## Supporting information


**Table S1**. Antibodies and recombinant proteins used in the microfluidic single‐cell phenotyping experiment.
**Figure S1**. Gating strategy used for the flow cytometric analysis of graft‐derived cells. Total cells were identified based on FSC‐A/SSC‐A parameters (a), followed by the selection of single cells (b) and exclusion of PI^+^ (dead) cells (c). Significant proportion of the cells were FAP‐ CD45‐ (d).
**Figure S2**. Flow cytometric characterization of cells isolated from LCA biopsies of the control group (mean ± SD; n = 7).
**Table S2**. Literature data* on the concentration of selected cytokines in the synovial fluid of uninjured knees.

## Data Availability

The data that support the findings of this study are available from the corresponding author upon request.

## References

[1] Agostinone P , Romandini I , Hantouly A , Al‐Sewaidi G , Oliver‐Welsh L , Veizi E , et al. Higher body mass index, postoperative bracing, narrower femoral notch, and anterior tibial tunnel positioning are independent risk factors for cyclops syndrome following anterior cruciate ligament reconstruction. J ISAKOS. 2026;18:101117. 10.1016/j.jisako.2026.101117 41985843

[2] Aloisi G , Goderecci R , Fidanza A , Cipolloni G , Calvisi V . Histological and clinical analysis of knee cyclops lesions. Acta Biomed. 2023;94(3):2023125. 10.23750/abm.v94i3.13953 PMC10308458 37326276

[3] Arner JW . Editorial commentary: risk factors of cyclops syndrome in quadriceps autograft anterior cruciate ligament reconstruction: more helpful data in weighing graft choice. Arthroscopy. 2023;39(6):1480–1482. 10.1016/j.arthro.2023.01.016 37147075

[4] Basalova N , Alexandrushkina N , Grigorieva O , Kulebyakina M , Efimenko A . Fibroblast activation protein alpha (FAPα) in fibrosis: beyond a perspective marker for activated stromal cells? Biomolecules. 2023;13(12):1718. 10.3390/biom13121718 PMC10742035 38136590

[5] Bradley DM , Bergman AG , Dillingham MF . MR imaging of cyclops lesions. Am J Roentgenol. 2000;174(3):719–726. 10.2214/ajr.174.3.1740719 10701615

[6] Chang Y , Lee JWN , Holle AW . The mechanobiology of fibroblast activation in disease. APL Bioengineering. 2025;9(2):021505. 10.1063/5.0272393 PMC12178607 40538750

[7] Darby IA , Laverdet B , Bonté F , Desmoulière A . Fibroblasts and myofibroblasts in wound healing. Clin Cosmet Investig Dermatol. 2014;7:301–311. 10.2147/CCID.S50046 PMC4226391 25395868

[8] Delaloye JR , Murar J , Vieira TD , Franck F , Pioger C , Helfer L , et al. Knee extension deficit in the early postoperative period predisposes to cyclops syndrome after anterior cruciate ligament reconstruction: a risk factor analysis in 3633 patients from the SANTI Study Group Database. Am J Sports Med. 2020;48(3):565–572. 10.1177/0363546519897064 31930921

[9] Docking S , Samiric T , Scase E , Purdam C , Cook J . Relationship between compressive loading and ECM changes in tendons. Muscles Ligaments Tendons J. 2013;3(1):7–11. 10.11138/mltj/2013.3.1.007 PMC3676165 23885340

[10] Eckes B , Nischt R , Krieg T . Cell‐matrix interactions in dermal repair and scarring. Fibrogenesis Tissue Repair. 2010;3(1):4. 10.1186/1755-1536-3-4 PMC2855519 20222960

[11] Farr M , Garvey K , Bold AM , Kendall MJ , Bacon PA . Significance of the hydrogen ion concentration in synovial fluid in rheumatoid arthritis. Clin Exp Rheumatol. 1985;3(2):99–104. 4017318

[12] Haydinger CD , Ashander LM , Tan ACR , Smith JR . Intercellular adhesion molecule 1: more than a leukocyte adhesion molecule. Biology. 2023;12:743. 10.3390/biology12050743 PMC10215618 37237555

[13] Huang KL , Wang LK , Tsai FM . TGF‐β signaling as a pathological continuum linking idiopathic pulmonary fibrosis and lung cancer. Cells. 2026;15(5):480. 10.3390/cells15050480 PMC12985053 41827913

[14] Jackson JW , Frederick C. Streich Jr, U , Pal A , Coricor G , Boston C , Brueckner CT , et al. An antibody that inhibits TGF‐β1 release from latent extracellular matrix complexes attenuates the progression of renal fibrosis. Sci Signaling. 2024;17(844):eadn6052. 10.1126/scisignal.adn6052 38980922

[15] Jacob M , Chang L , Pure E . Fibroblast activation protein in remodeling tissues. Curr Mol Med. 2012;12(10):1220–1243. 10.2174/156652412803833607 22834826

[16] Kambhampati SBS , Gollamudi S , Shanmugasundaram S , Josyula VVS . Cyclops lesions of the knee: a narrative review of the literature. Orthop J Sports Med. 2020;8(8):2325967120945671. 10.1177/2325967120945671 PMC7457408 32923503

[17] Kelly T , Huang Y , Simms AE , Mazur A . Chapter three—fibroblast activation protein‐α: a key modulator of the microenvironment in multiple pathologies. In: Jeon KW , editor. International review of cell and molecular biology. Academic Press; 2012. p. 83–116. 10.1016/B978-0-12-394308-8.00003-0 22608558

[18] Lattouf R , Younes R , Lutomski D , Naaman N , Godeau G , Senni K , et al. Picrosirius red staining: a useful tool to appraise collagen networks in normal and pathological tissues. J Histochem Cytochem. 2014;62(10):751–758. 10.1369/0022155414545787 25023614

[19] Lawson C , Wolf S . ICAM‐1 signaling in endothelial cells. Pharmacol Rep. 2009;61(1):22–32. 10.1016/s1734-1140(09)70004-0 19307690

[20] Li Y , Zhao J , Yin Y , Li K , Zhang C , Zheng Y . The role of IL‐6 in fibrotic diseases: molecular and cellular mechanisms. Int J Biol Sci. 2022;18(14):5405–5414. 10.7150/ijbs.75876 PMC9461670 36147459

[21] Linder A , Portmann K , Schlotheuber LJ , Streuli A , Glänzer WS , Eyer K , et al. Microfluidic approach to resolve simultaneous and sequential cytokine secretion of individual polyfunctional cells. J Vis Exp. 2024;205:66492. 10.3791/66492 38526129

[22] López De Padilla CM , Coenen MJ , Tovar A , De la Vega RE , Evans CH , Müller SA . Picrosirius red staining: revisiting its application to the qualitative and quantitative assessment of collagen type I and type III in tendon. J Histochem Cytochem. 2021;69(10):633–643. 10.1369/00221554211046777 PMC8504258 34549650

[23] Marzo JM , Bowen MK , Warren RF , Wickiewicz TL , Altchek DW . Intraarticular fibrous nodule as a cause of loss of extension following anterior cruciate ligament reconstruction. Arthroscopy. 1992;8(1):10–18. 10.1016/0749-8063(92)90129-y 1550639

[24] Murphy J , LaVigne C , Rush A , Pendleton A . Risk factors for the development of arthrofibrosis after anterior cruciate ligament reconstruction in children and adolescents. Orthopedics. 2024;47(4):e161–e166. 10.3928/01477447-20240520-04 38810127

[25] Nikolic I , Morici L . When healing turns fibrotic: exploring molecular mechanisms and therapeutic strategies for knee arthrofibrosis. Eur J Pharmacol. 2026;1017:178645. 10.1016/j.ejphar.2026.178645 41655683

[26] Noailles T , Chalopin A , Boissard M , Lopes R , Bouguennec N , Hardy A . Incidence and risk factors for cyclops syndrome after anterior cruciate ligament reconstruction: a systematic literature review. Orthop Traumatol Surg Res. 2019;105(7):1401–1405. 10.1016/j.otsr.2019.07.007 31405748

[27] Öztürk R . Cyclops syndrome following anterior cruciate ligament reconstruction: can relapse occur after surgery? World J Orthop. 2024;15(3):201–203. 10.5312/wjo.v15.i3.201 PMC10999971 38596191

[28] Paschos NK . Editorial commentary: personalized anterior cruciate ligament reconstruction and rehabilitation mitigate postoperative arthrofibrosis: prevention is the best approach. Arthroscopy. 2024;40(5):1700–1702. 10.1016/j.arthro.2023.12.022 38244022

[29] Pommerolle L , Beltramo G , Biziorek L , Truchi M , Dias AMM , Dondaine L , et al. CD206(+) macrophages are relevant non‐invasive imaging biomarkers and therapeutic targets in experimental lung fibrosis. Thorax. 2024;79(12):1124–1135. 10.1136/thorax-2023-221168 PMC11672011 39033028

[30] Portmann K , Linder A , Eyer K . Stimulation‐induced cytokine polyfunctionality as a dynamic concept. eLife. 2024;12:RP89781. 10.7554/eLife.89781 PMC11254380 39018359

[31] Portmann K , Linder A , Oelgarth N , Eyer K . Single‐cell deep phenotyping of cytokine release unmasks stimulation‐specific biological signatures and distinct secretion dynamics. Cell Rep Methods. 2023;3(7):100502. 10.1016/j.crmeth.2023.100502 PMC10391336 37533643

[32] Qian W‐J , Yan J‐S , Gang X‐Y , Xu L , Shi S , Li X , et al. Intercellular adhesion molecule‐1 (ICAM‐1): from molecular functions to clinical applications in cancer investigation. Biochim Biophys Acta Rev Cancer. 2024;1879(6):189187. 10.1016/j.bbcan.2024.189187 39317271

[33] Roebuck KA , Finnegan A . Regulation of intercellular adhesion molecule‐1 (CD54) gene expression. J Leukoc Biol. 1999;66(6):876–888. 10.1002/jlb.66.6.876 10614768

[34] Roman J . Fibroblasts—warriors at the intersection of wound healing and disrepair. Biomolecules. 2023;13(6):945. 10.3390/biom13060945 PMC10296409 37371525

[35] Steele H , Cheng J , Willicut A , Dell G , Breckenridge J , Culberson E , et al. TNF superfamily control of tissue remodeling and fibrosis. Front Immunol. 2023;14:1219907. 10.3389/fimmu.2023.1219907 PMC10351606 37465675

[36] Struglics A , Larsson S , Kumahashi N , Frobell R , Lohmander LS . Changes in cytokines and aggrecan ARGS neoepitope in synovial fluid and serum and in C‐terminal crosslinking telopeptide of type II collagen and N‐terminal crosslinking telopeptide of type I collagen in urine over five years after anterior cruciate ligament rupture: an exploratory analysis in the knee anterior cruciate ligament, nonsurgical versus surgical treatment trial. Arthritis Rheumatol. 2015;67(7):1816–1825. 10.1002/art.39146 25914389

[37] Suvarna K.S. LC , Bancroft JD . Bancroft's theory and practice of histological techniques. Elsevier; 2018.

[38] Theil C , Ackmann T , Gosheger G , Puetzler J , Moellenbeck B , Schwarze J , et al. Synovial fluid pH is as specific as synovial leukocyte count but less sensitive for the diagnosis of chronic prosthetic joint infection. J Orthop Traumatol. 2022;23(1):52. 10.1186/s10195-022-00672-5 PMC9675884 36402933

[39] Tran E , Chinnasamy D , Yu Z , Morgan RA , Lee CCR , Restifo NP , et al. Immune targeting of fibroblast activation protein triggers recognition of multipotent bone marrow stromal cells and cachexia. J Exp Med. 2013;210(6):1125–1135. 10.1084/jem.20130110 PMC3674706 23712432

[40] Tschumperlin DJ , Ligresti G , Hilscher MB , Shah VH . Mechanosensing and fibrosis. J Clin Invest. 2018;128(1):74–84. 10.1172/JCI93561 PMC5749510 29293092

[41] Usher KM , Zhu S , Mavropalias G , Carrino JA , Zhao J , Xu J . Pathological mechanisms and therapeutic outlooks for arthrofibrosis. Bone Res. 2019;7(1):9. 10.1038/s41413-019-0047-x PMC6433953 30937213

[42] Wang J , Ao Y . Analysis of different kinds of cyclops lesions with or without extension loss. Arthroscopy. 2009;25(6):626–631. 10.1016/j.arthro.2008.12.006 19501293

[43] Welling W . Return to sports after an ACL reconstruction in 2024—a glass half full? A narrative review. Phys Ther Sport. 2024;67:141–148. 10.1016/j.ptsp.2024.05.001 38749349

[44] Wynn TA , Vannella KM . Macrophages in tissue repair, regeneration, and fibrosis. Immunity. 2016;44(3):450–462. 10.1016/j.immuni.2016.02.015 PMC4794754 26982353

[45] Zou AE , Kongthong S , Mueller AA , Brenner MB . Fibroblasts in immune responses, inflammatory diseases and therapeutic implications. Nat Rev Rheumatol. 2025;21(6):336–354. 10.1038/s41584-025-01259-0 40369134

