# Supplementary material for: Patients with cyclops syndrome exhibit elevated synovial fluid interleukin‐6 (IL‐6) and tumor necrosis factor alpha (TNF‐α) levels and disorganized collagen remodelling within the lesion: A histological and molecular study
Source: J Exp Orthop. 2026 Oct 17;13(4):e70953. doi: 10.1002/jeo2.70953 (PMC13676164; doi:10.1002/jeo2.70953)
Supplement: Supplementary file 1 [file JEO2-13-e70953-s001.docx]

## Supplementary Material Section

## Patients with Cyclops Syndrome Exhibit Elevated Synovial Fluid IL-6 and TNF-α Levels and Disorganized Collagen Remodelling Within the Lesion: A Histological and Molecular Study

Luca Morici^1^, Philippe M. Tscholl^2*^, Eline L. Skau^3^, Klaus Eyer^3*^, Lukas Clerc^2^, Morgan Gauthier^2^, Ines Nikolic^1*^

^1^University of Geneva, Institute of Pharmaceutical Sciences of Western Switzerland, Geneva, Switzerland

^2^University Hospital of Geneva, Department of Orthopaedics and Trauma Surgery, Geneva, Switzerland

^3^Aarhus University, Faculty of Health Sciences - Department of Biomedicine, Aarhus, Denmark

*Corresponding authors

Ines Nikolic: [ines.nikolic@unige.ch](mailto:ines.nikolic@unige.ch)

Philippe M. Tscholl: [philippe.tscholl@hug.ch](mailto:philippe.tscholl@hug.ch)

Klaus Eyer: [eyerk@biomed.au.dk](mailto:eyerk@biomed.au.dk)

##### Microfluidic single-cell phenotyping using *DropMap* technology

For the study here, paramagnetic nanoparticles were functionalized with cytokine-specific capture antibodies, prepared separately for each target, and the final functionalized nanoparticle suspensions were combined in optimized ratios (IL-1β:TNF-α:IL-6:TGF-β = 2:1:1:1). Fluorescently labelled detection antibodies were added to the nanoparticles prior to cell encapsulation.

For these experiments, cells were thawed and incubated either with recombinant human IL-1β (50 ng/mL) for 4 h at 37°C and 5% CO₂ in ultra-low attachment plates (Corning), or without added stimulant. At the end of the incubation period, the cells were gently resuspended, washed with pre-warmed cell buffer, and centrifuged at 300 g for 10 min. Cells were then resuspended at a final concentration of 10⁶ cells/mL.

***Table S1*.** Antibodies and recombinant proteins used in the microfluidic single-cell phenotyping experiment

| **Antibodies** | | |
| --- | --- | --- |
| **Name** | **Supplier** | **Cat. Number** |
| IL-6 Monoclonal Antibody (MQ2-39C3), Biotin | Thermo Fisher | 13-7068-85 |
| IL-6 Monoclonal Antibody (MQ2-13A5), FITC | Thermo Fisher | 11-7069-81 |
| TNF-α Monoclonal Antibody (MAb1), biotinylated in-house | Thermo Fisher | 14-7348-85 |
| TNF-α Monoclonal Antibody (MAb11), PE | Thermo Fisher | 12-7349-81 |
| IL-1β Polyclonal Antibody, Biotin | PeproTech | 12-7349-81 |
| IL-1β Monoclonal Antibody, AF647 labelled in-house | PeproTech | 500-M01B |
| TGF-β Monoclonal Antibody (1D11), biotinylated in-house | Thermo Fisher | MA5-23795 |
| TGF-β Monoclonal Antibody (1D11), AF647 labelled in-house | Thermo Fisher | MA5-23795 |
| **Recombinant protein (for stimulation experiment)** | | |
| Human IL-1β Recombinant Protein | PeproTech | 200-01B |

##### Flow cytometry

***Figure S1*. Gating strategy used for the flow cytometric analysis of graft-derived cells**. Total cells were identified based on FSC-A/SSC-A parameters (a), followed by the selection of single cells (b) and exclusion of PI⁺ (dead) cells (c). Significant proportion of the cells were FAP^-^ CD45^-^ (d)

***Figure S2.*** Flow cytometric characterization of cells isolated from LCA biopsies of the control group (mean ± SD; *n* = 7).

| ***Tale S2***. Literature data* on the concentration of selected cytokines in the synovial fluid of uninjured knees | | | | |
| --- | --- | --- | --- | --- |
| **Cytokine** | **Values in SF of uninjured knees (median, pg/ml)** | **Method** | **Number of samples** | **Reference** |
| IL-1β | Below LLOQ | Multiplex human pro-inflammatory 7-plex immunoassay | 21 (39% women)  Age: 29±9.4 years | Larsson, S., A. Struglics, L.S. Lohmander, and R. Frobell, Surgical reconstruction of ruptured anterior cruciate ligament prolongs trauma-induced increase of inflammatory cytokines in synovial fluid: an exploratory analysis in the KANON trial. Osteoarthritis and Cartilage, 2017. 25(9): p. 1443-1451. |
| IL-6 | 0.24 (0.11-0.45) |  |  |  |
| TNF-α | 0.19 (0.10 – 0.29) |  |  |  |
| *Samples obtained in the course of the 5-year randomized controlled trial for acute ACL rupture: Knee Anterior Cruciate Ligament: Nonsurgical versus Surgical Treatment (KANON); SF: synovial fluid; LLOQ: lower limit of quantification | | | | |
